# Fabrication of Magnetically Actuated Fluidic Drug Delivery Device Using Polyvinyl Chloride Adhesive Stencils

**DOI:** 10.3390/mi9070358

**Published:** 2018-07-19

**Authors:** Hyun Kim, Jong-mo Seo

**Affiliations:** 1 Department of Electrical and Computer Engineering, Inter-University Semiconductor Research Center, Institute of Engineering Research, Seoul National University, Seoul 151-742, Korea; colatincan@snu.ac.kr or pepsitincan@gmail.com; 2 Department of Ophthalmology, Biomedical Research Institute, Seoul National University Hospital, Seoul 03080, Korea

**Keywords:** fluidic drug delivery device, polyvinyl chloride adhesive stencil, polydimethylsiloxane fabrication, magnetically actuated microfluidic device, controlled release rate

## Abstract

In this paper, a polydimethylsiloxane (PDMS) fabrication method is introduced. It eliminates the need for conventional fabrication methods, such as photolithography and etching. Only a series of oxygen plasma treatments, silanization, and polyvinyl chloride (PVC) adhesive stencils were used to develop multi-layer designs. The fabrication method was applied to fabricate a PDMS-based drug delivery device with an actively controllable, magnetically actuated valve. Above all, this fabrication method eliminated the use of a power-consuming pump. Fluidic substances were injected into the circular shaped primary chamber through a syringe. A secondary chamber, similar to the primary chamber’s structure but with a smaller radius and thinner membrane, was connected via a microchannel to regulate the amount released. When actuated with a permanent magnet for one second, the volume in the secondary chamber first depletes. As the magnet is removed, the valve closes. Subsequently, the primary chamber replenishes the secondary chamber. This process can be repeated until the primary chamber reaches a saturation state that can no longer inflate the secondary chamber. The device could release a few microliters per actuation. Various combinations of size and thickness of primary, and secondary chambers can realize release rate of desired amount.

## 1. Introduction

Therapeutic drug delivery requires the regulation of drug substances released into the system to maintain a plasma concentration to a level between Maximum Tolerated Concentration (MTC) and Minimum Effective Concentration (MEC). Conventional drug administration methods, systemic and local, however, fail to do so for various complexities: Adequate quantitative regulation, periodic and manual administration, etc. There has been an uptick in research of advanced drug delivery devices that can realize effective drug therapy [1,2,3] locally or systemically. Localized drug delivery devices require the ability to deliver exact amount of drug substances to desired sites at a desired time.

Recently, many localized drug delivery systems have been developed, seeking to realize on-demand temporal control. Most of these devices are polymer based and take advantage of their physical and chemical properties, such as degradation, to achieve temporal release. These passively controlled devices rely on biodegradability [4,5,6], diffusion [7], osmotic pressure [8], microfluidic channel designs [9], etc. Due to their releasing mechanism, drug release begins simultaneously with the implantation of the device. Some studies show that actively controlled drug delivery devices that are responsive to external stimuli have advantages over passive devices, in realizing on demand temporal control [10]. A typical actively controlled drug delivery device consists of a pump, a valve, a reservoir and an actuation mechanism that controls the release rate [11]. However promising these systems may be, several challenges still remain. Micrometer-scale pump, channel, reservoir, and valve fabrication technologies have been developed, yet integrating them to build a single system is challenging.

Microvalves and micropumps are mandatory components of an on-demand drug delivery system. A variety of active microvalves and micropumps triggered by electrostatic [12], electromagnetic [13], magnetic [14], pneumatic [15], bimetallic [16], and piezoelectric [17] actuation have been developed using traditional Micro-Electromechanical Systems (MEMS) fabrication methods. A crucial point of a microvalve is its initial mode: normally opened [18,19,20,21] or normally closed [22,23,24]. The initial mode is an important factor in drug delivery systems for it determines the total power consumption of the device. Implantable systems rely on an on-chip battery source for operation. Since the battery occupies most of the device’s spatial area, lesser power consumption is crucial for further miniaturization. Micropumps, also essential components of drug delivery systems, require multiple layers of complex fabrication and thus are expensive and difficult to miniaturize. Therefore, novel designs pursue easily fabricated, less expensive, and minutely controllable micropumps.

**Figure 1:**
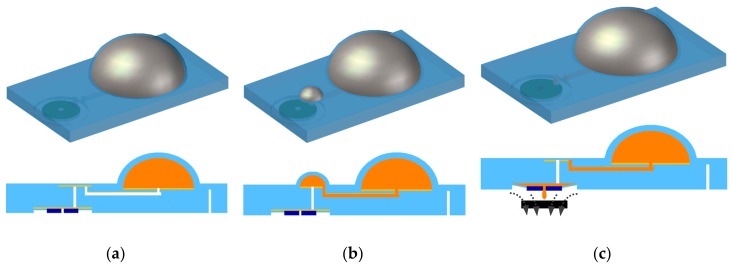
Schematic of the proposed drug delivery device. (**a**) Fluidic drug substances are injected into the device through a syringe. The primary chamber inflates. (**b**) The secondary chamber is subsequently inflated as the substances from the primary chamber travel through the microchannel. (**c**) The magnetic polydimethylsiloxane (PDMS) membrane is deflected using a permanent magnet releasing the secondary chamber’s volume first. The magnet is removed and the valve is closed. (**b**) and (**c**) procedures can be repeated until the primary chamber reaches saturation.

In this paper, new polydimethylsiloxane (PDMS) fabrication methods are introduced and utilized to develop an actively controlled fluidic drug delivery device. Polyvinyl Chloride (PVC) adhesive sheets were designed and cut using a Diode Pumped Solid State (DPSS) laser. PVC stencils were used to manipulate PDMS polymerization [25] to realize easy fabrication of microchannels non-lithographically. Moreover, PVC stencils were also used to develop a zero volume chamber that can act as a pump. Zero volume chambers initially have zero volume. They can be inflated and deflated in response to the internal pressure. The nature of these chambers obviate the need of a power consuming pump; thus on demand release of fluidic substances can be realized only by regulating the magnetically actuated microvalve. Normally closed initial mode of the microvalve was achieved to minimize power consumption. Figure 1 displays the schematic of the device. Experiments were all conducted using deionized water (DI) water to easily deduce the amount released at each actuation.

## 2. Design and Fabrication

**Figure 2:**
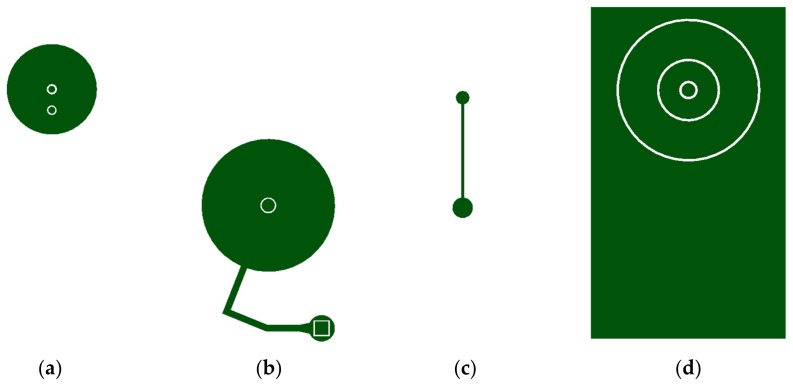
Polyvinyl chloride (PVC) stencils used in the fabrication process. (**a**) Stencil for the secondary chamber. The smaller circles are inlet and outlet for the secondary chamber (inlet = circle away from the center, outlet = circle in the center). (**b**) Stencil for the primary chamber (outlet = Small circle in the center, inlet = rectangle, bolting microchannel = thin fragment). (**c**) Stencil that fabricates the microchannel that connects the secondary and primary chamber. (**d**) Stencil for the magnetically actuated microvalve (outlet of the device = smallest circle, nickel-PDMS stencil = smaller circular band stencil, deflection membrane = larger circular band stencil).

The conventional PDMS microstructure fabrication process includes PDMS patterning via soft lithography [26] and etching [27,28]. This process produces rectangular shaped microstructures with fixed width and height. To develop zero volume chambers, a new fabrication method is necessary. Here, a non-lithographic approach was taken. A combination of oxygen plasma treatment, silanization, and polyvinyl chloride (PVC) stencil masks were used to develop each component. The fabrication procedure requires four different PVC stencil designs; one for each component. Figure 2 shows four different PVC stencil designs. Utilizing these stencils, the proposed method introduces a novel PDMS fabrication method that produces adjustable PDMS structures that can change shape and size in response to the internal pressure.

PVC stencils in Figure 2 were all acquired using a DPSS laser system (Samurai UV Marking System, DPSS Laser Inc., Santa Clara, CA, USA). This system setting was fixed to 100% laser power, frequency of 20.00 Hz, mark speed of 100.00 mm/s, and pulse width of 20 μs. The accuracy of this system was analyzed by cutting stencils with length 2 cm and widths ranging from 100 μm to 1 mm. The system had high precision with an average of approximately 20 μm discrepancy to the desired dimensions. Moreover, the smallest feature this system could acquire was approximately 80 μm.

### 2.1. Selective Bonding and Inflatable Chamber Fabrication

PDMS has different surface properties before and after polymerization: Hydrophilic and hydrophobic. The inherent hydrophobicity of fully polymerized PDMS limits its usage in various microfluidic applications. Moreover, when pre-cured PDMS is dispensed over fully polymerized PDMS and cured, the two permanently bond with no separable boundary. To fabricate the inflatable structure selective bonding of surfaces between layers is necessary. Here, a combination of oxygen plasma treatment and silanization was adopted. Silanization develops an anti-stiction barrier between the first and second layer while oxygen plasma treatment promotes bonding of the two layers. To perform selective bonding, a layer of PDMS was spin-coated, cured and silanized. The designed PVC stencil was pasted on the layer and treated with oxygen plasma at a flow rate of 5 sccm and 5 W of radio frequency (RF) power for 45 s. Then, the stencil was removed and PDMS was dispensed over the first layer and cured. The process developed a PDMS double layer with detached areas equivalent to the stencil shape.

**Figure 3:**
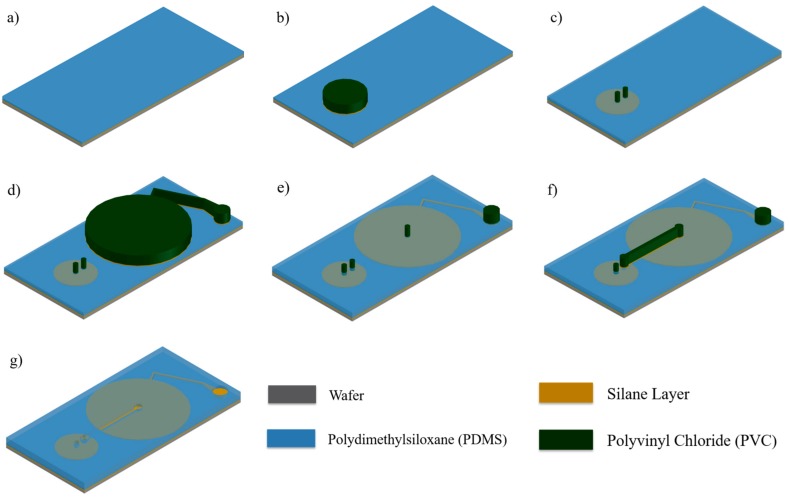
Inflatable/deflatable chamber fabrication steps. (**a**) Spin coating PDMS on silanized wafer. (**b**) Paste secondary chamber stencil on silanized PDMS and treat with oxygen plasma. (**c**) Remove chamber portion of the stencil leaving the inlet and outlet stencils. Then, spin coat PDMS. (**d**) Paste primary reservoir stencil on silanized PDMS and treat with oxygen plasma. (**e**) Remove chamber portion leaving the inlet and outlet stencils. Then, spin coat PDMS. (**f**) Align and paste microchannel stencil on silanized PDMS and treat with oxygen plasma. (**g**) Finally, spin coat PDMS and cure. Remove remaining stencils leaving three holes: inlet, outlet and microchannel. Yellow portions depict the detached area. In other words, the detached area illustrates the inflating and deflating areas.

The fabrication process of the drug delivery device starts with exposing the wafer to silane vapor by evaporating trichloro (1H, 1H, 2H, 2H-tridecafluoro-n-octyl) silane in a vacuum chamber. Prior to silanization, the wafer was oxygen plasma treated. Silanization undertakes several roles in the proposed fabrication method. This process allows not only easy removal of PDMS structures from the wafer but also secure fixation of PVC adhesive stencils. Then, PDMS solution is prepared by mixing a curing agent (Sylgard^®^ 184B, Dow Corning, Midland, MI, USA) to a PDMS liquid pre-polymer (Sylgard^®^ 184A, Dow Corning, Midland, MI, USA) in a 1:10 weight ratio and degassed using a centrifuge. 3 mL of this mixture is dispensed on a wafer using a pipette and spin coated with a series of spin speeds of 500 rpm for 5 s, 1000 rpm for 15 s, and 500 rpm for 5 s. The layer is cured at 100 °C for 5 min resulting in a thickness of approximately 100 μm (Figure 3a). This first layer of PDMS comprises the inflatable membrane of the secondary chamber. Selective bonding was performed on this first layer with the stencil in Figure 2a. After oxygen plasma treatment (Figure 3b), only the chamber portion of the stencil was removed; the inlet and outlet portions were still pasted on the layer. Then, PDMS was dispensed over the layer and cured (Figure 3c). After curing, selective bonding was again performed on the second layer using the stencil in Figure 2b. Similar to the first selective bonding process, the inlet and outlet portions of the stencil remained on the surface (Figure 3e).

### 2.2. Flow Rate Regulating Microchannel

The wafer now has two inflatable chambers with two inlet and two outlet stencils (Figure 3e). The outlet of the primary chamber and the inlet of the secondary chamber are connected via a microchannel. This microchannel’s cross-sectional area determines the flow rate of fluidic substances pumped from the primary chamber to the secondary chamber. This microchannel is also non-lithographically fabricated using the PVC stencil in Figure 2c. The primary chamber’s outlet stencil and the secondary chamber’s inlet stencil are removed. As the two are removed, PDMS coated over the stencil are removed, too. As a result, two holes with equal shape and size to the stencils are left on the surface. The microchannel stencil is aligned to cover the two holes and pasted. PDMS is spin coated and cured (Figure 3f). Peeling off the microchannel stencil completes the fabrication of this microchannel (Figure 3g).

### 2.3. Magnetically Actuated Microvalve

**Figure 4:**
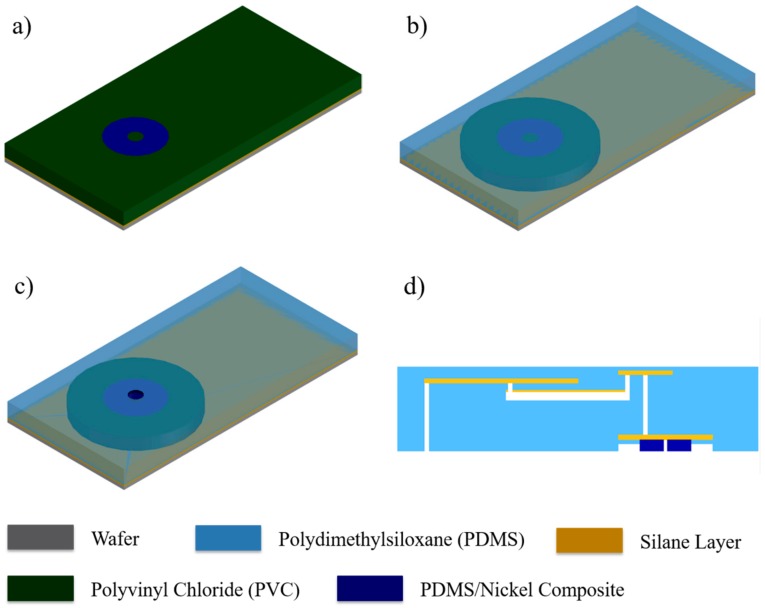
Magnetically actuated micro-valve fabrication. (**a**) Paste PVC stencil on silanized wafer. Remove circular band and fill with nickel-PDMS composite. (**b**) Remove rectangular stencil and spin coated PDMS over the wafer. (**c**) remove center circle PVC stencil leaving a hole in the center. (**d**) Cross-sectional view of the drug delivery device after integration with chamber layer, valve layer and slab of PDMS.

Magnetically actuated microvalve fabrication begins with a new silanized wafer. The microvalve stencil (Figure 1d) is directly pasted on the wafer (Figure 4a). The smaller circular band is removed from the wafer and filled with a mixture of PDMS and nickel micro-particles (APS 2.2~3.0 micron, Thermo Fisher Scientific, Waltham, MA, USA) in a 1:1 ratio. The composite is poured over the stencil and scraped off with a blade (Figure 4b). Nickel-PDMS is then cured to have the equivalent shape and thickness to the stencil. Here, to ensure uniformity of the particles in the mixture, a strong magnet was placed under the wafer until the composite fully polymerized. After polymerization, the rectangular stencil is removed from the wafer and PDMS was coated. Finally, the smallest circular stencil is removed making an outlet hole. Fluidic substances filled in the primary chamber is released into the medium through this outlet hole.

### 2.4. Integration via Plasma Bonding

The primary chamber, secondary chamber, and magnetically actuated valve fabrications are already completed. Simply bonding a slab of PDMS in between the two layers then completes the proposed drug delivery device. First, the slab is bonded to the chamber layer. The slab of PDMS has two holes punctured. One is aligned with the inlet of the primary chamber and the other with the outlet of the secondary chamber. PDMS bonding is implemented by oxygen plasma treatment. To ensure bonding, the device was heated at 60 °C for 30 min. Lastly, the valve layer is bonded to the device, here, a circular stencil was used to execute selective bonding. Figure 4d is the cross-sectional image of the completed device.

### 2.5. Experimental Setup

A Teflon tube with an outer diameter of 1.57 mm is connected to the inlet of the device. Using a syringe pump (NE-1010 Programmable Single Syringe Pump, New Era Pump Systems Inc., New York, NY, USA), desired amount of DI water is injected into the device. The device is left to reach an equilibrium between the primary and secondary chambers. A strong permanent magnet is placed right beneath the device to deflect the membrane releasing substances out of the device. Then, the magnet is removed closing the device. Before each actuation, the device should be left untouched for at least 5 min for the chambers to reach an equilibrium. The released amount at every actuation is measured by weighing the fluidic substance. Converting the weight to volume with the density of DI water (0.997 g/mL at room temperature), the released amount in μL can be calculated.

## 3. Results

### 3.1. Surface Modification and Selective Bonding

**Figure 5:**
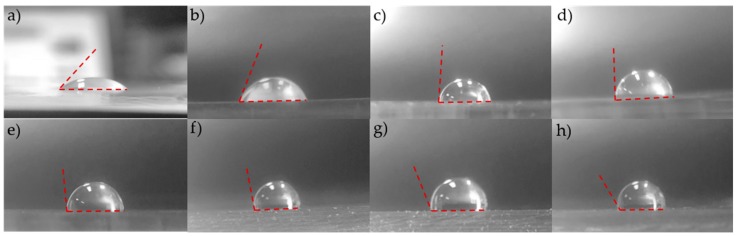
Water drop contact angles on PDMS surfaces treated with different radio frequency (RF) power oxygen plasma before silanization. (**a**) 5 W, (**b**) 10 W, (**c**) 30 W, (**d**) 50 W, (**e**) 80 W, (**f**) 100 W, (**g**) 150 W, (**h**) 200 W.

Selective bonding proposed in this paper utilizes a combination of oxygen plasma treatment and silanization. Figure 5 shows water drop contact angles on PDMS surfaces treated with 8 different RF powers during oxygen plasma treatment prior to silanization: 5, 10, 30, 50, 80, 100, 150, and 200 W. PDMS surfaces treated with low power oxygen plasma treatment, 5 to 10 W of RF power, produced hydrophilic surfaces. High power treatments, however, produced hydrophobic surfaces.

**Figure 6:**
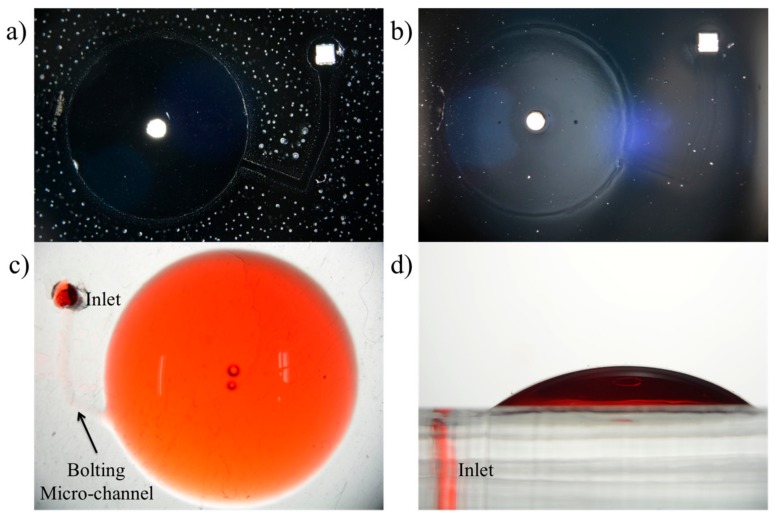
Surface treatment of PDMS by oxygen plasma and silanization. (**a**) Silanized PDMS surface was covered with the primary reservoir stencil (Figure 1b) and treated with oxygen plasma. (**b**) PDMS is dispensed over the treated surface and cured. Patterned surface can clearly be visualized. (**c**) Inflated primary chamber with inlet and bolting microchannel indicated with an arrow. (**d**) Side view of the inflated primary chamber.

The patterned surface can be visualized in Figure 6a due to the difference in hydrophilicy of silanized and oxygen plasma treated surfaces. PDMS was dispensed on the patterned surface and cured. Figure 6b shows the PDMS double layer where detached areas can also be clearly visualized. Figure 6c,d shows images of the inflated chamber injected with dyed DI water. A syringe was connected at the inlet and DI water was injected manually to show successful implementation of selective bonding.

### 3.2. Microchannel Fabrication

**Figure 7:**
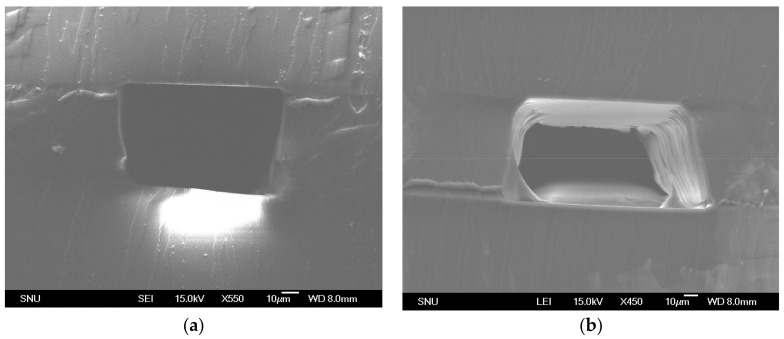
(**a**) and (**b**) shows cross-sectional area of PDMS microchannels developed using PVC stencils.

Microchannel fabrication using PVC stencils manipulate PDMS polymerization conditions [25]. PDMS polymerization inhibition could be observed only over PVC stencils. Depending on the thickness deposited on the stencil, the degree of PDMS polymerization inhibition can be determined. Due to the polymerization inhibition of PDMS, the peeling off motion of the stencil can tear off on the boundary where fully polymerized PDMS and polymerization inhibited PDMS meet, which is directly over the stencil boundary. Figure 7a,b shows cross-sectional scanning electron microscope (SEM) images of the microchannels made with the proposed method. Depending on the degree of PDMS polymerization inhibition, the microchannels showed overcut or vertical cut profiles. In most cases, there are no residue or debris left in the manufacturing process. However, in a few cases, liquid PDMS may leak into the crevice between the PDMS substrate and the stencil. Figure 7b shows leakage of PDMS. Despite leakage in a few cases, the proposed method presents a simple and fast microchannel fabrication alternative that does not require soft lithography. This method can be used for fast prototyping microchannels with resolution as high as a few tens of micrometers.

### 3.3. Chamber Maximum Volume

**Figure 8:**
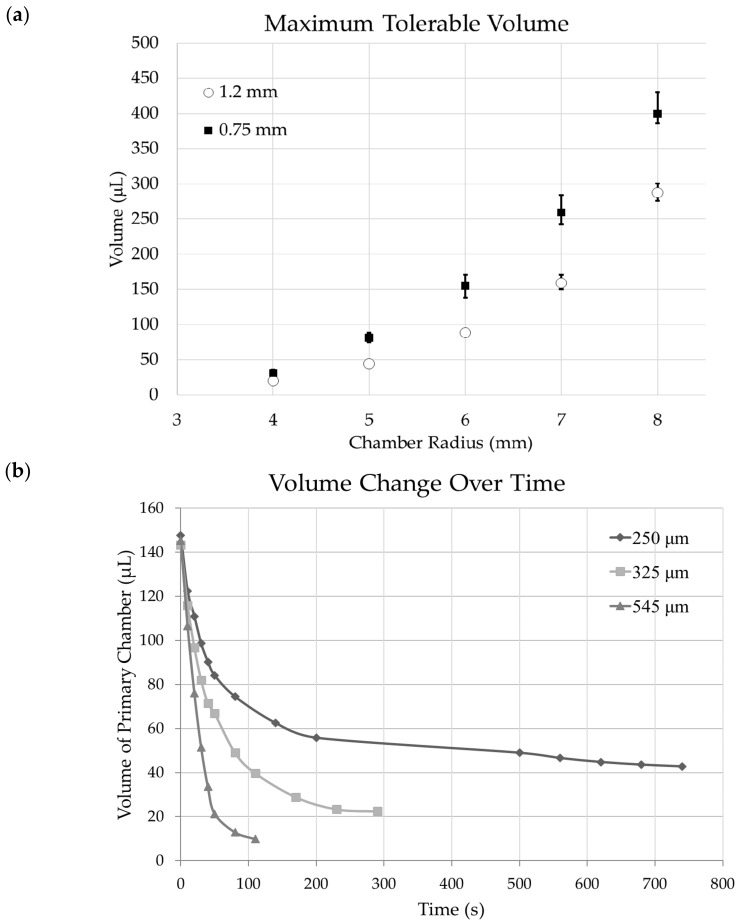
(**a**) Charts depict maximum volume the inflatable reservoir can handle with respect to the radius of the chamber, thickness of the membrane, and the width of the bolting microchannel. (**b**) Release rate of the inflatable chamber with three thickness variations; 250 μm, 325 μm, and 545 μm.

There are many factors that determine the maximum tolerable volume of the primary chamber. The chamber is designed to have a narrow inflatable channel that connects the inlet with the chamber. Fluidic substances are injected into the chamber using a syringe, manually, through this channel. The channel also works as a bolting mechanism prohibiting substances from flowing out into the medium through the inlet. When substances are injected into the chamber using a syringe, there is enough power to inflate the thin bolting channel. Substances travel through the channel and enters the chamber consequently inflating the chamber. As the substances inflate the chamber, the internal pressure of the chamber increases. When the pressure exerted by the chamber is large enough to inflate the bolting channel, substances will flow out of the chamber through the inlet. Figure 8a shows maximum tolerable volume of chambers with radius ranging from 4 mm and 8 mm and membrane thickness of 250 μm. Two different bolting channel widths, 1.2 mm and 750 μm, were tested to experimentally acquire the maximum tolerable volume. Surplus of substances were injected into the chamber and waited until leakage of substances stopped.

### 3.4. Fluid Release

**Figure 9:**
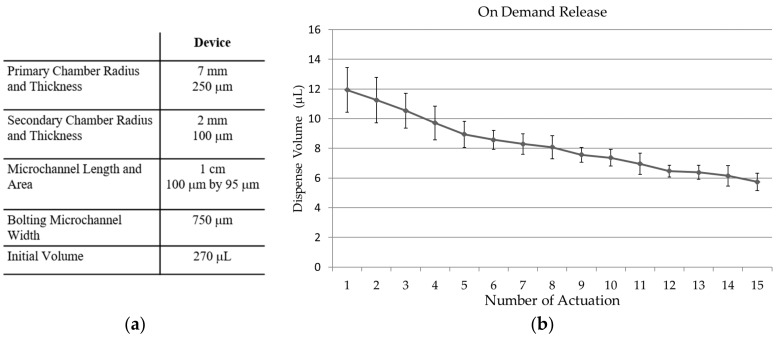
(**a**) Dimensions and initial conditions of the tested drug delivery device. (**b**) shows a chart of on demand release of the device described in (**a**). The device was actuated 15 times using a strong neodymium magnet.

**Figure 10:**
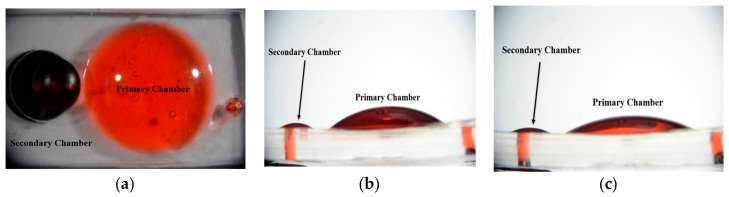
Image of the actual device. (**a**) Top view of the device. The primary chamber and the secondary chamber is labeled. (**b**) Side view of the device with primary chamber filled with 270 μL of dyed DI water. (**c**) Side view of the device with primary filled with approximately 190 μL of dyed DI water after 8 actuations.

Dimensions of the drug delivery device is described in Figure 9a. On demand DI water release of the device with primary and secondary chamber is shown in Figure 9b. Actuation was implemented with a strong neodymium magnet for 1 s each time. The x-axis of the chart in Figure 9b is the number of actuations. The device was actuated 15 times. The device has a secondary chamber’s membrane thickness and size: 100 μm and radius 2 mm. The primary chamber’s size was designed to be 7 mm with membrane thickness 250 μm. The bolting microchannel was 750 μm in width and the volume was approximately 270 μL. On demand release of the device was tested 10 times. The device started with an average of 11.94 μL and decreased down to 5.74 μL after 15 actuations. With a thicker primary chamber, the release rate decreased rather rapidly while a thinner chamber decreased slower. Figure 10 shows images of the actual device. Figure 10a is a side view image of the device when the primary chamber was filled with red dyed DI water of approximately 270 μL. Figure 10c is a side view image of the device after 8 actuations of the device. The primary chamber was left with 190 μL. The difference in volume of the secondary chamber of Figure 10b,c can be visualized by the difference in height.

DI water release rate of the primary chamber without the secondary chamber is shown in Figure 8b. Three devices with chambers each with different thicknesses were analyzed: 250 μm, 325 μm, and 545 μm. The release amount depends on the thickness of the chamber membrane, and cross-sectional area of the microchannel. Each device was injected with approximately 145 μL of DI water for testing. The bolting inlet channel was 1.2 mm thick and the cross-sectional area of the microchannel was fixed to 100 μm by 95 μm. For all three devices, the release rate rapidly decreased and reached nearly zero in a matter of minutes. Moreover, the devices did not completely deplete until the release rate reached nearly zero. The release rate of the device with 250 μm membrane reached zero approximately after 780 s. The chamber was left with 42.8 μL. Similarly, the release rate of 325 μm and 545 μm thick membrane devices reached zero after 290 s and 110 s, leaving 22.4 μL and 9.8 μL. Consequently, the thinner the chamber membrane was, the longer it took for the release rate to reach zero.

## 4. Conclusions

In this paper, a PDMS fabrication method utilizing PVC stencils to develop a drug delivery device is introduced. This fabrication process does not use any of the conventional MEMS fabrication methods. PVC stencils prepared with the DPSS laser system was used with oxygen plasma treatment and silanization to implement selective bonding and etching. Exclusion of MEMS fabrication methods were realized to devise simple fabrication methods that require neither clean rooms nor garments. Along with simple fabrication method introduced in this paper, the drug delivery device proposed in this paper achieved minimal power consumption, taking advantage of PDMS’s flexibility and tensile strength. Inflatable drug chamber releases substances out as the chamber deflates. With only one chamber, the release rate rapidly decreased. Therefore, two chambers with different thickness and size were integrated to prevent rapid release of substances. Moreover, the release mechanism also used the flexibility and physical characteristics of PDMS. Magnetically actuated, on demand release of the device gradually decreased after a total of 15 actuations. The first and the 15th release amount differed by approximately 5 μL. More combinations of thicknesses and sizes of primary and secondary chambers may facilitate desired release rates.
